# Mechanisms of melanocyte death in vitiligo

**DOI:** 10.1002/med.21754

**Published:** 2020-11-17

**Authors:** Jianru Chen, Shuli Li, Chunying Li

**Affiliations:** ^1^ Department of Dermatology Xijing hospital, Fourth Military Medical University Xi'an Shannxi China

**Keywords:** autoimmunity, death, melanocyte, oxidative stress, vitiligo

## Abstract

Vitiligo is an autoimmune depigment disease results from extensive melanocytes destruction. The destruction of melanocyte is thought to be of multifactorial causation. Genome‐wide associated studies have identified single‐nucleotide polymorphisms in a panel of susceptible loci as risk factors in melanocyte death. But vitiligo onset can't be solely attributed to a susceptive genetic background. Oxidative stress triggered by elevated levels of reactive oxygen species accounts for melanocytic molecular and organelle dysfunction, a minority of melanocyte demise, and melanocyte‐specific antigens exposure. Of note, the self‐responsive immune function directly contributes to the bulk of melanocyte deaths in vitiligo. The aberrantly heightened innate immunity, type‐1‐skewed T helper, and incompetent regulatory T cells tip the balance toward autoreaction and CD8^+^ cytotoxic T lymphocytes finally execute the killing of melanocytes, possibly alarmed by resident memory T cells. In addition to the well‐established apoptosis and necrosis, we discuss several death modalities like oxeiptosis, ferroptosis, and necroptosis that are probably employed in melanocyte destruction. This review focuses on the various mechanisms of melanocytic death in vitiligo pathogenesis to demonstrate a panorama of that. We hope to provide new insights into vitiligo pathogenesis and treatment strategies by the review.

## 1 INTRODUCTION

**Figure 1:**
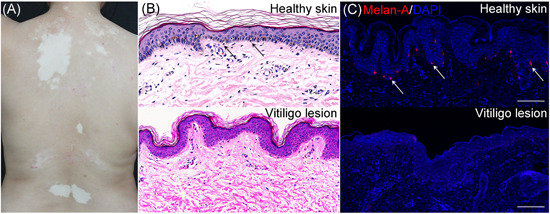
Clinical and pathological manifestation of vitiligo. A, Vitiligo is characterized by chronic depigmentation and milk‐white lesion in the skin. B, Representative images of normal melanocytes in the stratum basale of healthy control and an absence of melanocytes in vitiligo skin. The sections stained with hematoxylin and eosin (×400, black arrowheads in the inset: Melanocytes). C, Healthy skin with normal quantity of melanocytes in contrast to vitiligo lesional skin being devoid of melanocytes on account of melanocyte destruction, as detected by immunofluorescence. Melanocytes in the epidermis were stained with antibodies to Melan‐A (red). Nuclei were counterstained with 4′,6‐diamidino‐2‐phenylindole (blue) (scale bar = 100 μm) (white arrowheads in the inset: Melanocytes) [Color figure can be viewed at wileyonlinelibrary.com]

**Figure 2:**
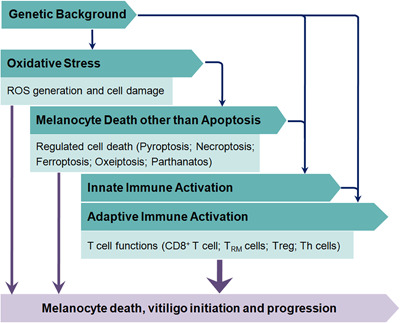
A road map for melanocyte in vitiligo. The figure briefly illustrates the main contents of melanocyte death triggers and the logical relation between them in vitiligo. ROS, reactive oxygen species; Th cells, T‐helper cells; Treg: regulatory T cell; T_RM_, resident memory T cell [Color figure can be viewed at wileyonlinelibrary.com]

Vitiligo is an autoimmune disease featured as chronic depigmentation and milk‐white lesion in the skin with a prevalence of 0.5% of the general population (Figure 1A). Although not accompanied by disturbing senses like pruritus or pain, vitiligo might negatively affect people's self‐esteem and even cause anxiety or depression, as the disfiguring spots hamper their social interaction.^1^ What's worse, the curative effects of existent treatments for vitiligo are not optimistic. Moderately effective therapies like phototherapy (psoralen combined with ultraviolet A, narrowband ultraviolet B [NB‐UVB]), topical treatment (corticosteroid, calcineurin inhibitors), and systemic treatment (corticosteroid) are still practically and financially burdensome.^2^ The wide prevalence of this chronic disease and unsatisfying therapeutic effect underscore the need to further explore vitiligo pathogenesis. Pathologic destruction of melanocyte is the major foci in vitiligo onset and development, as depicted in Figure 1B,C, and how to forestall and even reverse the process has always been an emphasis of vitiligo basic research. Although both segmental vitiligo (SV) and nonsegmental vitiligo (NSV) exhibit melanocyte destruction, whether the destruction of melanocytes in SV is attributed to the autoimmune response or inherent cellular abnormalities remains obscure.^3^ Considering that NSV is more prevalent^4^ with its pathogenesis more thoroughly studied, we discuss mechanisms of melanocyte death within the scope of NSV in the following parts and do not distinguish between “NSV” and “vitiligo”. Recent years have seen unprecedented growth in the understanding of vitiligo pathogenesis，which results from a sizeable set of studies focusing on dissecting the molecular mechanism involved in vitiligo melanocyte destruction. And in this review, we summarize these molecular mechanisms and their interplay in the perspective of melanocyte death in the spectrum of genetic predisposition, oxidative stress‐related subversion, and immunological onslaught. The same causal mechanisms by no means apply to all cases, and different pathogenetic mechanisms might work together, ultimately leading to the same clinical result. We also briefly discuss some death modalities putatively harbored in melanocyte destruction (Figure 2). Besides, the cutting‐edge technologies assisting vitiligo diagnosis as well as current status and future perspectives of therapeutic approaches to prevent melanocytes from death is also included in the hope of providing sparks for future research.

## 2 GENETIC BACKGROUND

Vitiligo is thought to be of multifactorial causation. Specifically, the concordance of generalized vitiligo in monozygotic twins is approximately 23%.^5^ Vitiligo patients’ relatives also suffer an approximately 6‐ to 18‐fold risk for vitiligo than the masses.^5^ Additionally, the epidemiological study revealed that single‐nucleotide polymorphisms (SNPs) in multiple risk genes are related to the arising of vitiligo.^6^ Concerning all these facts, the genetic background has been emphasized to be a significant endogenous factor in vitiligo pathogenesis. In recent years, extensive genome‐wide associated studies shed light on more than 40 susceptibility loci, including *MC1R*, *TYR*, *IFIH1*, *CD44*, *CD80*, *GZMB*, *HLA‐A*, *XBP1*, *CAT*, and *MTHFR*.^7^, ^8^ Of the identified candidate loci, more than half are involved in immunoregulation, T‐cell receptor repertoire, and immune cell‐associated apoptosis, indicating the critical effect of aberrant immune function in vitiligo pathogenesis.^8^ The extending list of susceptibility loci might form a scale, to which the results of patients’ gene sequencing could be compared, to evaluate the patients’ “vitiligo risk score". The evaluation might help to optimize vitiligo diagnosis, personalized treatment, and prognosis.

Polymorphisms in *TYR* and *HLA‐A* (see below) direct the immune system to target melanocytes. *TYR* encodes tyrosinase, which is not only an enzyme catalyzing melanogenesis but an autoantigen presented by human leukocyte antigen‐A on the surface of the melanocyte. Tyrosinase epitopes are presented to immature T cells by Langerhans cell to activate an autoimmune response, ultimately induce apoptosis of melanocytes.^9^ Intriguingly, the effects of two European‐derived missense variants *R402Q* (SNP rs1126809) and *S192Y* (SNP rs1042602) have been solidified in diminishing vitiligo susceptibility by reducing the thermostability of tyrosinase protein, conducing to the decline of tyrosinase epitopes presented, which weakens tyrosinase autoantigen availability.^6^, ^10^, ^11^ *HLA‐A* encodes the α chain of HLA class I histocompatibility antigen. Jin et al.^12^ performed DNA sequence analysis and identified the high‐risk allele HLA‐A*02:01:01:01.^12^ The underlying causal mechanism might be an SNP haplotype (rs12206499) spanning transcriptional regulatory region downstream of *HLA‐A*, which instigates the upregulation of HLA‐A*02:01:01:01 messenger RNA (mRNA), ultimately precipitating autoreactive T‐cell to recognize melanocyte antigens and to destruct melanocytes.^13^ Moreover, there exists significant epistasis between *HLA‐A* SNP rs12206499 and *TYR* SNP rs1393350,^10^ implying the interplay of the two polymorphisms in promoting vitiligo susceptibility.

*FAS* and *FASLG* polymorphisms are also critical members of vitiligo SNPs. The Fas–Fas ligand (FasL) system (encoded by *FAS* and *FASLG*) controls the final step of cell apoptosis, and functional polymorphisms of the two genes might perturb their transcriptional activities. The *FAS* polymorphisms (*FAS*‐1377A>G) and *FASLG* SNP rs78037977 are reported to be associated with higher vitiligo risk.^8^, ^14^ Variants at many other loci like *CAT* (encodes catalase to detoxify hydrogen peroxide [H_2_O_2_]),^15^ *IFIH1* (functions as pattern recognition receptor to activate innate immune response),^16^ and *GZMB* (encodes granzyme B to mediate cytotoxic T cell‐induced apoptosis)^17^ also confer increased predisposition to vitiligo onset. Nevertheless, consensus on precise mechanisms whereby most of the candidate genes confer vitiligo risk has not been established, except their epidemiological association with vitiligo susceptibility. After all, the candidate loci merely provide a genetic background, and many other factors such as oxidative stress and derailed immune function must be included to elucidate vitiligo pathogenesis.

## 3 OXIDATIVE STRESS

Oxidative stress is considered one of the most crucial initiators in vitiligo occurrence,^18^ despite consensus on an exact etiology of vitiligo has not been established. Other factors like metabolism possibly engender melanocyte deaths, yet examples backing them up are sparse.^19^ Oxidative stress is disturbed redox homeostasis characterized by the imbalance of prooxidants and antioxidants. Oxidative stress in tissue and cell always results from excessive reactive oxygen species (ROS). ROS embody H_2_O_2_, hydroxyl radical, hypochlorous acid, and hydroperoxyl radical.^20^ In the past several decades, ROS has been proved to be an important second messenger molecule, nevertheless, a high concentration of ROS is implicated in murdering melanocyte in all aspects, including undermining DNA, lipid, protein, and their metabolites structurally and functionally.^21^, ^22^ Furthermore, ROS‐induced oxidative stress widely instigates aberrant organelle functions, derails metabolism pathways and compromises defensive mechanism against the onslaught of oxidative agents. A growing body of evidence has provided a plausible connection between oxidative stress and deficiency of keratinocyte, melanocyte stem cell, and extracellular microenvironment.^4^, ^23^, ^24^ All the factors, as mentioned above, may help inform our understanding of melanocyte destruction in vitiligo. As for this part, we try to illustrate the panorama of oxidative stress and its association with melanocyte obliteration.

### 3.1 Source of ROS

**Figure 3:**
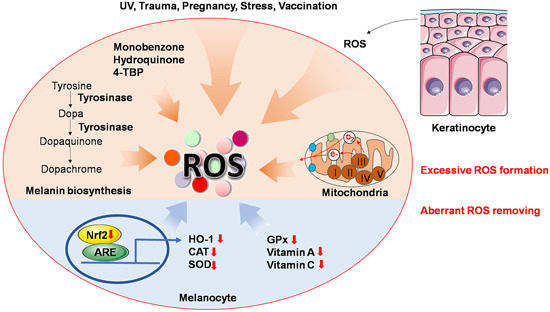
Source of ROS. Accumulation of ROS can be generated by either excessive formation or inadequate scavenging. Environmental stimuli like UV, trauma, pregnancy, stress, and vaccination promote ROS formation. External phenols including monobenzone, hydroquinone and 4‐TBP also give rise to ROS as known. Melanin biosynthesis and energy metabolism in mitochondria physically yield ROS. Neighboring keratinocytes also reportedly transfer ROS to melanocytes. With respect to inadequate scavenging of ROS, downregulated Nrf2 pathway and enzymic or nonenzymic antioxidant agents should be attributable. 4‐TBP, *4‐tertiary butyl‐phenol*; ARE, antioxidant response element; CAT, catalase; GPx, glutathione peroxidase; HO‐1, heme oxygenase‐1; Nrf2, nuclear factor E2‐related factor 2; ROS, reactive oxygen species; SOD, superoxide dismutase [Color figure can be viewed at wileyonlinelibrary.com]

The generation of highly enriched ROS to which melanocytes are subject can be attributed to two reasons, excessive formation and inadequate scavenging (Figure 3). Overproduction of ROS is partly triggered by stimuli from the environment, such as ultraviolet (UV) radiation, cytotoxic chemical agents, trauma, pregnancy, stress, and vaccination.^4^, ^25^ The generation of ROS is an instant intracellular response when the cell is exposed to UV irradiation.^26^ Phenols, including monobenzone, hydroquinone, and *4‐tertiary butyl‐phenol*, are structurally comparable to melanogenesis substrate tyrosine, thus, capable of covalently binding to tyrosinase, which proceeds to the generation of ROS.^27^, ^28^, ^29^ Excessive ROS might also simultaneously come from melanogenesis and mitochondrial energy metabolism. In the process of melanosynthesis, ROS generation ensues from the conversion of dopa to dopaquinone and dopaquinone to dopachrome.^30^ Moreover, melanogenesis is an energy‐consuming procedure, indicating the need for large amounts of adenosine triphosphate (ATP). Biosynthesis of ATP per se is accompanied by the generation of ROS in mitochondria. Mitochondria are suggested to be a dominating source of ROS due to their manipulation of oxidative stress‐related aging and apoptosis.^4^ Under cellular stress, electrons escape from the respiratory chain to react with molecular oxygen thus generating superoxide anions and the secondary ROS.^31^ In an immortalized perilesional vitiligo melanocyte line PIG3V, suppression on *PPARGC1A* (encoding peroxisome proliferator‐activated receptor γ coactivator‐1α) expression was attenuated owing to downregulated microRNA (miR)‐211. The expression of *PPARGC1A* gives rise to the ROS overproduction.^32^ Interestingly, keratinocytes appear to transfer H_2_O_2_ into neighboring cells including melanocytes, which can be significantly reduced by adding catalase to intercellular media.^33^

Other than excessive formation, aberrant ROS removing mechanisms also account for a plethora of ROS in the epidermis. Downregulated enzymic or nonenzymic defense against oxidants in the epidermis, such as the incapacity of catalase, glutathione peroxidase, and decrease of vitamins A and C underpin the accumulation of ROS.^25^, ^34^, ^35^ Another impaired antioxidant pathway in both melanocyte and keratinocyte,^36^ nuclear factor E2‐related factor 2 (Nrf2)‐antioxidant response element (ARE)/heme oxygenase‐1 (HO‐1), also merits mention. Once cellular oxidative stress occurs, the critical transcription factor Nrf2 regulates expression of antioxidant genes by binding to ARE sequence and fuels the transcription of extensive downstream antioxidative enzymes such as HO‐1, catalase, NADH quinone oxidoreductase 1 and superoxide dismutase.^37^, ^38^ Jian et al.^35^, ^39^ highlighted that the Nrf2‐ARE pathway deficiency and concomitant antioxidant incapacity confer a persuasive mechanism for melanocytes’ sensitivity towards oxidative stress in vitiligo patients.

### 3.2 Macromolecules compromised by ROS

Accumulated ROS‐induced oxidative stress in the epidermis of vitiligo patients has been validated. About the impact of oxidative stress on vitiligo occurrence, one plausible hypothesis states that oxidative stress compromises macromolecules in epidermis melanocytes, which precipitate the loss of functional melanocytes. A series of studies have enrolled macromolecule oxidation products such as advanced oxidative protein products, advanced glycosylation end‐products, malondialdehyde, and 8‐hydroxy‐2’‐deoxyguanosine as diameters of evaluating disease progression in vitiligo patients, because levels of these products are directly associated with extension, duration, and disease activity of vitiligo.^40^, ^41^

Noteworthily, as a result of chronic oxidative stress stimuli, melanocyte DNA undergoes persistent damage.^41^ After that, the cell might be orchestrated into cellular senescence featuring irreversible cell proliferation arrest.^42^, ^43^ The process is governed by a strong cell cycle checkpoint mechanism wherein activation of p53, p16, and p21 are indispensable,^44^ and extensive melanocyte cell‐cycle arrest might accentuate the obliteration. Furthermore, ROS‐induced lipid peroxidation might also lead to mitochondrial deterioration, which will be discussed exclusively later. In this context, senescent melanocyte exhibits a special “senescence‐associated secretory phenotype (SASP)” and expresses a multitude of factors including interleukin (IL)‐6, cyclooxygenase, extracellular matrix metalloproteinases (MMPs) and insulin‐like growth factor binding protein.^45^, ^46^ Part of those secretions might even recruit immune cells to scavenge the “senescent melanocyte,” termed as “senescence‐surveillance” (from antitumor immunity),^47^ which is a potential way of melanocyte destruction. It deserves noting that the procedure is a vicious spiral, signals released by the cell via SASP induce senescence of surrounding cells in a paracrine manner, and of themselves in an autocrine manner, thus autonomously enhancing cellular senescence.

### 3.3 Mitochondrial dysfunction

**Figure 4:**
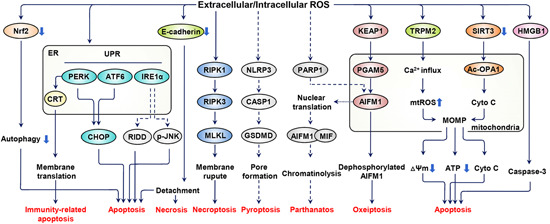
Oxidative stress leads to multiple death modalities through various pathways in melanocyte. Excessive ROS hyperactivate the UPR to trigger endoplasmic‐reticulum‐associated melanocytic apoptosis. Translocation of CRT from endoplasmic reticulum under oxidative stress adds to melanocyte immunogenicity. Mitochondria also participate in ROS‐related apoptosis through forming MOMP. Besides, redundant ROS give rise to deficient Nrf2 pathway, generation of HMGB1 and E‐cadherin diminution, which underlie melanocyte apoptosis. Of note, oxidative stress also evokes other modalities of melanocytic death, like necroptosis, necrosis, oxeiptosis, and probably pyroptosis and parthanatos. Some pathways surmised to exist are drawn in dotted lines. Ac‐OPA1, acetylated optic atrophy 1; AIFM1, apoptosis‐inducing factor mitochondrion‐associated 1; ATF6, activating transcription factor 6; CASP1, caspase 1; CHOP, CAAT/enhancer‐binding protein homologous protein; CRT, calreticulin; ER, endoplasmic reticulum; GSDMD, gasdermin D; IRE1α, inositol‐requiring enzyme‐1α; KEAP1, kelch‐like ECH‐associated protein 1; MIF, macrophage migration inhibitory factor; MLKL, mixed lineage kinase domain‐like protein; MOMP, mitochondrial outer membrane permeabilization; mtROS, mitochondrial ROS; Cyto *C*, cytochrome *c*; NLRP3, NLR family pyrin domain containing 3; Nrf2, nuclear factor E2‐related factor 2; PARP1, poly(ADP‐ribose) polymerase 1; PERK, PRKR‐like ER kinase; PGAM5, PGAM family member 5; p‐JNK, phosphorylated c‐Jun N‐terminal kinase; RIDD, regulated IRE1‐dependent messenger RNA decay; RIPK1, receptor‐interacting serine/threonine kinase 1; RIPK3, receptor‐interacting serine/threonine kinase 3; SIRT3, sirtuin 3; HMGB1, high‐mobility group box 1; TRPM2, transient receptor potential cation channel subfamily M member 2; UPR, unfolded protein response; ΔΨm, the mitochondrial membrane potential [Color figure can be viewed at wileyonlinelibrary.com]

Mitochondria are not only origin of ROS, but also victims in the flood of oxidative stress (Figure 4). Governing energy supply, aging, and apoptosis of healthy cells, mitochondria are closely linked to the fate of cells, accordingly, mitochondrial impairment is bound to affect melanocyte survival.^4^, ^25^, ^48^ Kang et al.^49^ ascertained that melanocyte demise can be interpreted as a transient receptor potential cation channel subfamily M member 2 (TRPM2)‐mediated Ca^2+^ influx into the cytoplasm and further mitochondrial‐dependent apoptosis. In the upstream, oxidative stress induce the production of TRPM2 by amplifying DNA demethylation.^49^ Activated sirtuin 3‐optic atrophy 1 axis is also substantiated to exacerbate mitochondrial damage, which is documented as an indispensable part of oxidative stress‐triggered melanocyte apoptosis.^50^ ROS‐associated mitochondrial dysfunction, which is illustrated by mitochondrial outer membrane permeabilization, mitochondrial membrane potential loss, and subsequent apoptosis, have also been reported in other cell lines.^51^, ^52^, ^53^ And the intricate molecular mechanisms of mitochondrial‐associated apoptosis are reviewed in Reference 50.

Aberrant lipid composition and compromised intactness of the respiratory chain in mitochondria are also parts of oxidativestress‐induced impairment. As extremely activated radicals, ROS potently damage biological macromolecules like lipid, reportedly causing lipid peroxidation, less cardiolipin, and more cholesterol in distribution.^54^, ^55^ Associated with that, the housing of the electron transport chain on lipid is also modified, which is a biochemical basis of mitochondrial impairment.^56^ Due to oxidative stress‐derived deficiency in assembly and intrinsic vulnerability to oxidative injury, mitochondrial complex, especially complex I,^32^, ^57^ exhibits dysfunction and further induces more ROS production thus forming a vicious spiral ^58^ to destroy melanocyte, and to elevate the sensibility to proapoptotic stimuli.^59^

A series of research has been conducted about the effect of several medicines on improving mitochondrial homeostasis. Corresponding results document that baicalein and berberine can restore mitochondrial dysfunction thus promoting melanocyte viability, mainly by decreasing intracellular ROS, downregulating proapoptotic protein levels and facilitating mitochondrial biogenesis.^60^, ^61^ To conclude, a growing body of evidence has been proving mitochondria as a crux in oxidative stress‐mediated melanocyte obliteration, which is also presenting potential targets for clinical therapy to concur vitiligo.

### 3.4 Endoplasmic reticulum abnormality

Except being an important site, whereby proteins are synthesized, folded, modified, and transported, the endoplasmic reticulum (ER) is also an organelle sensing cellular stress and regulating the survival or perishment of the cell. Among the various exogenous and endogenous perturbations, like UV irradiation, redox stress, infections and chemical agents, oxidative stress is a vital factor for ER dysfunction in vitiligo (Figure 4).^21^, ^62^ Morphologically, dilated ER in melanocytes from vitiligo patients was observed,^63^ and the effect of ROS on ER dilation can't be excluded. Recently, a series of research substantiates that ER subverted by ROS even induces melanocytes apoptosis. Cellular responses to perturbation like excessive ROS in ER function, termed “ER stress,” is characterized as aberrantly folded protein in the ER lumen, which further triggers unfolded protein response (UPR). Subtoxic UPR is required for restoring normal cellular function, but excessive and prolonged UPR might be a cell death mechanism.^64^

UPR comprises three parallel signaling branches: Inositol‐requiring enzyme 1α‐X‐box‐binding protein 1 pathway, activating transcription factor 6 pathway and PRKR‐like ER kinase‐eukaryotic translation initiation factor 2α pathway. Continuously activated by oxidative stress‐induced ER stress, the three branches might work independently or in concert to elicit apoptosis (Figure 4). ^65^ Highly enriched molecular mechanisms link UPR and apoptosis (like regulated IRE1 dependent decay and phosphorylated Jun amino‐terminal kinase pathways),^65^ however, scarcely are there researches to thoroughly substantiate the specific underlying mechanism in vitiligo. Oxidative stress‐triggered Ca^2+^ disturbance in the ER may also evoke UPR and the final apoptosis.^66^ In oxidative stress‐affected melanocyte and keratinocyte, the release of UPR‐regulated cytokines and chemokines (IL‐6, IL‐8, C‐X‐C motif chemokine ligand [CXCL] 12, CC chemokine ligand [CCL] 5) also recruits immune cells and indirectly devotes to melanocyte destruction.^21^, ^67^ Noteworthily, UPR‐induced autophagy is speculated to stimulate the production of exosomes and soluble inflammatory signals, which are proinflammatory and autoimmunity‐promoting.^62^ But autophagy is essentially a protective response in the face of cellular stress,^68^ and defective autophagy adds to melanocytic sensitivity towards oxidative stress.^69^ For this assessment, we think autophagy is more of an antiapoptotic mechanism, whose deficiency might lead to melanocytic apoptosis in the pathogenesis of vitiligo.

The effect of ROS also extends to other macromolecular in ER. Calreticulin (CRT), a ubiquitous ER protein modulating intracellular Ca^2+^ homeostasis, is manifested to be positively related to lesional area and duration of vitiligo in patients. The putative mechanism is that oxidative stress propels CRT redistribution from the ER lumen to cell membrane thus elevating the immunogenicity of stressed melanocyte and induces apoptosis (Figure 4). Furthermore, CRT stimulates the generation of proinflammatory cytokines that recruit immunocytes to eliminate melanocytes.^25^, ^70^ ER is an organelle standing at the crossing of cellular biosynthesis and survival, and is sensitive to various stimuli especially oxidative stress. The central role ER plays in melanocyte destruction of vitiligo pathogenesis can never be overemphasized.

### 3.5 Keratinocyte‐mediated melanocyte loss

Keratinocyte numerically dominates the cell composition of the epidermis, it also interacts with melanocyte directly.^71^, ^72^ Cumulative research indicate that keratinocyte is not only an innocent bystander in melanocyte destruction, but a vital nexus in vitiligo pathomechanism. Keratinocyte is a mediator of oxidative stress detriment, secreting cytokines to recruit autoreactive T cells and interfering signal transduction in melanocytes which consequently induce melanocyte destruction.^73^, ^74^ Intriguingly, Ca^2+^ and membrane‐associated thioredoxin reductase (TR) synergistically plays a kindred role in melanocytes undermining. Vitiliginous keratinocyte reportedly manifests defective Ca^2+^ uptake kinetics, which underlies an elevated‐Ca^2+^ microenvironment, thus TR, a pivotal enzyme modulating free‐radical defense and melanin biosynthesis, is allosterically inhibited and culminates in defense breakdown and melanogenesis inhibition in melanocytes.^75^, ^76^ Shi et al.^77^ substantiated that oxidative stress triggers overexpression of miR‐25 in keratinocytes and melanocytes, a microRNA that epigenetically suppresses the expression of microphthalmia‐associated transcription factor thus leading to a dysfunctional response to oxidative stress due to decreased activity of antioxidative enzymes and impaired transportation of melanosome. Moreover, the elevated level of miR‐25 undermines the paracrine of growth factors like stem cell factor and basic fibroblast growth factor from keratinocytes, both of which support melanocyte melanogenesis and survival. Similar to miR‐25, high‐mobility group box 1 (HMGB1) released from keratinocytes stimulated by oxidative stress may also put melanogenesis and melanocyte survival in peril, mainly by downregulating the activity of melanogenetic molecular like gp100, and initiating the apoptosis signaling, respectively.^78^ Parenthetically, HMGB1 translocating from melanocyte nucleus to cytoplasm in oxidative stress could also induce apoptosis through suppressing Nrf2 expression.^79^

Apart from the aforementioned factors, keratinocyte‐dependent recruitment of immunocyte under oxidative stress is also implicated in melanocyte destruction. IL‐17 is reported to be upregulated in the skin/serum of vitiligo patients.^80^ Excessive IL‐17 promotes IL‐1β secretion from keratinocytes via the ROS–NOD‐like receptors (NLR) family pyrin domain containing 3 (NLRP3)‐caspase pathway, thus coordinating local inflammation and attracts and activates adaptive immunocytes.^58^ Stimulated by ROS, secretion of CXCL‐16 and trans‐presentation of IL‐15 in keratinocytes recruits and activates CD8^+^T cell, respectively, and mediate melanocyte‐specific cytotoxic response.^73^, ^81^ ROS‐induced release of ATP from keratinocyte might not only stimulate neighboring keratinocytes to generate CXCL9 to chemoattract CD8^+^T cell, but also trigger caspase‐3‐mediated melanocyte apoptosis.^82^ Besides, in the context of ROS, elevated TRPM2 expression gives rise to NLRP3 inflammasome activation. In the downstream, CD8^+^T cell migration and effector function are both potentiated by IL‐1β/IL‐1R signaling.^83^ Hence keratinocyte is decisive for recruiting autoreactive immunocytes to kill melanocytes, as elaborated in the last several emblematic studies.

In vitiligo patients, melanocytes in the basal layer of unaffected zone epidermis are 25% less compared with healthy control, and melanocytes detachment from the basal layer into the upper layer has also been observed, which is termed as “transepidermal loss”.^84^ The results described herein concur to delineate a pathogenetic scenario that melanocyte detach from the basal layer was followed by transepidermal migration and eventually, melanocyte death occurs.^85^, ^86^ Specifically, unlike keratinocyte–keratinocyte adhesion, melanocytes are born poorly attached to surrounding keratinocyte due to less E‐cadherin expressed on surface.^85^ Under oxidative stress, elevated Src kinase expression and altered lipid raft arrangement disrupt protein interaction of the β‐catenin with E‐cadherin on melanocyte surface, ultimately weaker melanocyte–keratinocyte adhesion and enhanced melanocyte detachment.^87^, ^88^ And the shedding of E‐cadherin seems to be precipitated also by type‐1 cytokines like interferon (IFN)‐γ and tumor necrosis factor (TNF)‐α through activated MMP‐9, according to a recent publication.^89^ Furthermore, the detachment disrupts fibronectin adhesion‐mediated apoptosis suppression, and migrating to the upper layer of epidermis indicates more onslaught from deleterious stimulations like UV, both of which intensify melanocytes apoptosis and necrosis.^90^, ^91^ Taken together, oxidative stress amplifies the innate deficiency of melanocyte–keratinocyte adhesion and causes melanocyte transepidermal loss.^92^ Keratinocyte‐related mechanisms summarized above might work in parallel or hierarchical fashion to trigger or aggravate melanocyte obliteration in vitiligo pathogenesis (Figure 4).

### 3.6 Potential therapeutic approaches to ameliorate oxidative stress

Inferring from the factors mentioned above, we think it's high time to find effective therapeutic approaches to ameliorate oxidative stress in vitiligo patients to prevent melanocytes from destruction. NB‐UVB is reported to relieve oxidative stress in vitiligo patients.^93^ But the evidence remains to be solidified considering redox‐related indicators in erythrocyte are far from portraying epidermis redox status in this study. Aside from phototherapy, a sizeable set of antioxidants have been validated to protect melanocytes from oxidative stress‐induced mortality in vitro or/and in vivo. Polyphenols are preferred owing to their antioxidant activities. Polyphenols compounds including Ginkgo biloba extracts, apigenin and baicalin have all been documented in vitro to activate the Nrf2 pathway to counteract oxidative stress as yet.^61^, ^94^, ^95^ Quercetin also possesses anti‐inflammatory and antioxidative capacity, albeit not studied in vitiligo treatment.^96^ Apart from polyphenols, an array of organics, such as glycyrrhizin, afzelin, 6‐Shogaol, simvastatin, aspirin, also demonstrated their antioxidative ability by targeting Nrf2 pathways.^97^, ^98^, ^99^, ^100^, ^101^ Vitamin D can ameliorate ROS in melanocytes and restore their viability in oxidative stress through activating Wnt/β‐catenin pathway.^102^ Surprisingly, adipose tissue secretome might normalize H_2_O_2_‐ and UV‐induced oxidative stress in fibroblasts, despite its relatively weak efficacy in melanocytes.^103^ Inorganic substances like molecular hydrogen, palladium and platinum also target Nrf2 pathways in vitro,^104^, ^105^ but they are still far from therapeutically adopted considering the poor operability of molecular hydrogen and allergenic activity of metals.

Albeit extensively studied and applied, the antioxidants mentioned above are still not recommended as monotherapy in clinical practice. The main reason is the limited efficacy of antioxidants observed in clinical trials due to small numbers of participants and heterogeneity in the design of the trials. Thus, clinical trials with larger amounts of patients and more standardized compatibilities of the antioxidants should be implemented in the future. Moreover, we can conclude that highly enriched studies on counteracting oxidative stress are centered on scavenging the ROS that have been generated. But research on how to retard the excessive generation of ROS in the upstream are still sparse. Future studies might focus on restricting redundant ROS emission in melanocyte metabolism to control oxidative stress forwardly.

For now, innovative strategies for drug delivery, namely, nano‐drug delivery systems,^106^ are under development as reviewed in Reference 107. Particles like liposomes, niosomes, microemulsions and nanoparticles carrying drugs permeate into the deeper skin layer and play as the drug repository to slow down the drug release and reduce the frequency of treatments.^107^ Several in vitro experiments have been carried out utilizing the nano‐drug delivery system, the preliminary results of which are optimistic.^108^, ^109^ Additionally, precise drug delivery might also be employed to promote drug accumulation around the target cell and protect melanocyte from death. The affinity between receptors and ligands are utilized in this strategy. Vectors with ligand activity carry drugs that could target melanocyte‐specific receptors like melanocortin‐1 receptor and endothelin receptor.^106^ The combination of efficient drug delivery systems and efficacious drugs would upgrade vitiligo therapies to a higher level.

## 4 OTHER MELANOCYTE DEATH MODALITIES

Concluding from what was mentioned earlier, we find that apoptosis is the most extensively documented way of melanocyte demise, with few melanocytes undergo necrosis. But forms of cell death are not merely restricted to apoptosis and necrosis. Cells may die from accidental cell death (ACD) or regulated cell death (RCD). ACD, like necrosis, is biologically uncontrolled, whereas RCD (apoptosis, necroptosis, pyroptosis, oxeiptosis, ferroptosis, parthanatos, etc.) involves precise signaling cascade and molecular mechanisms, despite rare studies on nonapoptotic melanocyte death in vitiligo etiology. Hence, we introduce several nonapoptotic RCD forms and their possible connection with melanocyte destruction in vitiligo, in hope of providing potential directions for future studies (Figure 4).

### 4.1 Pyroptosis

Pyroptosis is mechanistically distinct from other forms of cell death with gasdermin D (GSDMD) and caspase‐1/4/5/11 activation as its defining feature.^110^ Upon being stimulated by damage‐associated molecular patterns molecules (DAMPs), pathogen‐associated molecular patterns molecules or altered homeostasis, caspases are activated to cleave the downstream GSDMD. GSDMD fragment with membrane pore‐forming activity yields plasma membrane rupture, cytosolic content release, and concurrent cell swelling and lysis.^111^ Pyroptotic cell releases a formidable number of cytokines and proteins that may act as “find me” or “eat me” signals. Recently, pyroptosis has been found to participate in target cell death induced by cytotoxic lymphocytes through granzyme‐A‐cleaved gasdermin B.^112^ These results raise the interesting possibility in vitiligo, that pyroptosis might partly contribute to ROS‐ or cytotoxic T lymphocytes (CTLs)‐induced melanocyte death and the ensuing immunological outcomes.

### 4.2 Necroptosis

Necroptosis is a programmed form of necrosis, featured by receptor‐interacting serine/threonine kinase (RIPK) 3‐mixed lineage kinase domain‐like pseudokinase (MLKL) pathway.^113^ It can be triggered by various stimuli, for example, activation of toll‐like receptors (e.g., TLR3 and TLR4),^114^ death receptors (e.g., Fas),^115^ and adhesion receptors.^116^ Besides, previous research reported mitochondrial events like the production of mitochondrial ROS and the presence of mitochondrial permeability transition pore might trigger necroptosis.^117^, ^118^ Our laboratory has disclosed that oxidative stress precipitates melanocyte necroptosis via RIPK1‐RIPK3‐MLKL pathway (article accepted by *Journal of Investigative Dermatology*) in vitiligo. And further studies should be carried out to elucidate the immune consequence of melanocyte necroptosis.

### 4.3 Ferroptosis

Ferroptosis is an iron‐ and lipotoxicity‐dependent form of RCD, characterized by dysmorphic small mitochondria with decreased crista and condensed outer membranes in the absence of hallmarks of apoptosis or necroptosis.^107^, ^119^ A panel of vital factors like inactivation of glutathione peroxidase,^107^ p53 elevation,^120^ ROS accumulation and lipid peroxidation of membranes in ferroptosis^107^ is also shared by aforementioned melanocyte demise. Additionally, ferroptosis is reported to partake in IFN‐γ‐associated cell destruction, and that IFN‐γ could lead to melanocyte death is well‐established in vitiligo pathogenesis.^121^, ^122^ But whether ferroptosis is a participant in melanocyte destruction is undefined.

### 4.4 Oxeiptosis

Oxeiptosis is a ROS‐induced noninflammatory RCD. This novel kind of cell death is independent of apoptotic or pyroptotic caspases, necroptosis, autophagy and ferroptosis.^123^ Oxeiptosis is mediated by kelch‐like ECH‐associated protein 1 (KEAP1)‐PGAM family member 5 (PGAM5)‐apoptosis‐inducing factor mitochondrion‐associated 1 (AIFM1) pathway. KEAP1 is responsible for sensing and quantification of ROS. In low ROS concentration, KEAP1 activates Nrf2 as a cellular protectant, whereas in high ROS concentration, KEAP1 might release PGAM5 and interact with AIFM1 thus initiating the death signaling.^123^ We have found that oxeiptosis existed in the perilesional skin of vitiligo patients and ROS‐treated melanocytes manifested oxeiptosis (unpublished data). We surmise that self‐antigen exposure might be the immunological consequence of melanocyte oxeiptosis, despite oxeiptosis reportedly does not cause inflammatory reaction.^124^ The exact effect of melanocyte oxeiptosis on vitiligo pathogenesis remains to be explicated.

### 4.5 Parthanatos

Parthanatos is a caspase‐independent RCD activated by oxidative stress‐induced DNA damage.^125^ In parthanatos, DNA base injured by oxidative stress triggers poly polymerase‐1 (PARP‐1) hyperactivation. Overactivated PARP‐1 synthesizes PAR polymer to elicit the translocation of AIFM1 from the mitochondria to the nucleus, and the AIFM1 recruits macrophage migration inhibitory factor (MIF) to cleave genomic DNA into large fragments.^126^ Parthanatos is widely deployed in stroke, myocardial infarction, neurodegenerative diseases, and ROS‐induced injuries.^126^ It deserves noting that MIF concentrations were also found to be higher in serum and lesional skin of vitiligo patients.^127^, ^128^ Thus, whether parthanatos is instrumental in the oxidative stress‐related disease, vitiligo, might be explored in the future. And inhibiting parthanatos to make melanocyte step back from the brink of parthanatotic cell death might be well pursuing.

**Figure 5:**
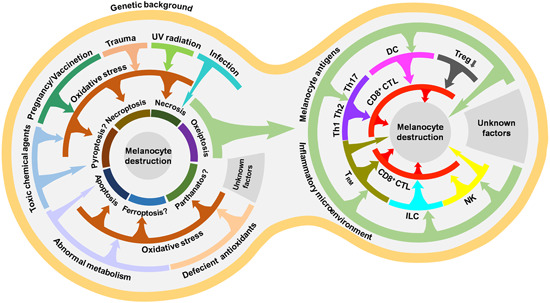
Overview of factors triggering melanocyte death in vitiligo. In the predisposing genetic background, environmental stimuli, aberrant metabolism and deficient antioxidants indirectly cause several possible death modalities of a fraction of melanocytes through ROS, though minor direct death‐triggering pathways exist. Outcomes of these melanocyte deaths, antigens exposure and inflammatory microenvironment, confer the majority of melanocyte demise via immune cells breaching the self‐tolerance. CD8^+^CTL, CD8^+^ cytotoxic T lymphocytes; DC, dendritic cell; ILC, innate lymphoid cells; NK, natural killer cells; Th1, T‐helper cell 1; Th17, T‐helper cell 17; Th2, T‐helper cell 2; Treg, regulatory T cell; T_RM_, resident memory T cell [Color figure can be viewed at wileyonlinelibrary.com]

So far, we have introduced the genetic background of vitiligo, oxidative stress‐induced melanocyte destruction, and other putative nonapoptotic regulated cell death modalities. On one hand, they provide evidence for the death of minority melanocytes in vitiligo. On the other hand, they bring about leakage of dead cell antigens and generation of the inflammatory microenvironment to trigger the immunity against melanocyte or “immunogenic cell death (ICD)” (a term in tumor immunity describing exposure or release of endogenous signaling from tumor cell rendering their death immunogenic).^113^ The ICD is the final form of most of the melanocytes which are killed in vitiligo. Thus, we employed a chart to illustrate that the genetic background of vitiligo, oxidative stress‐induced melanocyte destruction, and other putative nonapoptotic regulated cell death modalities that dictate melanocyte demise as well as pave the road for the following immunological consequences, the death of more melanocytes (Figure 5).

## 5 INNATE IMMUNE ACTIVATION

**Figure 6:**
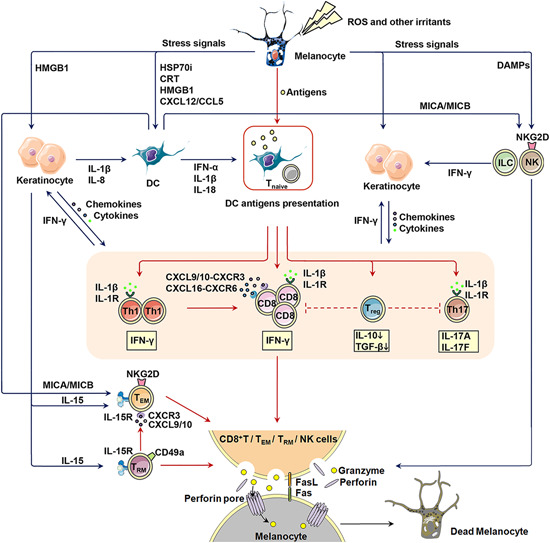
Aberrantly activated immune response in vitiligo. Outlined in the figure are members and their reciprocal relationship of the aberrantly activated innate and adaptive immunity in vitiligo pathogenesis. The interaction between innate immune cells are drawn in blue long‐tail arrow, and the interaction between adaptive immune cells in red. CCL5, CC chemokine ligand 5; CRT, calreticulin; CXCL12, C‐X‐C motif chemokine ligand 12; CXCL16, C‐X‐C motif chemokine ligand 16; CXCL9/10, C‐X‐C motif chemokine ligand 9/10; CXCR3, C‐X‐C motif chemokine receptor 3; CXCR6, C‐X‐C motif chemokine receptor 6; DAMP, damage‐associated molecular patterns molecule; DC, dendritic cell; Fas/FasL, Fas/Fas ligand; HMGB1, high‐mobility group box 1; HSP70i, inducible heat shock protein 70; IFN‐α, interferon‐α; IFN‐γ, interferon‐γ; IL‐10, interleukin‐10; IL‐15, interleukin‐15; IL‐15R, interleukin‐15 receptor; IL‐17A, interleukin‐17A; IL‐17F, interleukin‐17F; IL‐1R, interleukin‐1 receptor; IL‐1β, interleukin‐1β; IL‐8, interleukin‐18; ILC, innate lymphoid cells; MICA/MICB, major histocompatibility complex class I chain‐related protein A and B; NK, nature killer cells; NKG2D, natural killer group 2D; PRR, pattern recognition receptors; ROS, reactive oxygen species; T_EM_, effector memory T cells; TGF‐β, transforming growth factor‐β; Th1, T‐helper cells; Th17, T‐helper cell 17; TLR, toll‐like receptors; T_naive_, naïve T cells; Treg, regulatory T cells; T_RM_, resident memory T cells [Color figure can be viewed at wileyonlinelibrary.com]

In skin reside cells from nearly every division of the immune system, from macrophages, nature killer (NK) cells, and innate lymphoid cells (ILC) to CTL, regulatory T cells (Treg) and resident memory T (T_RM_) cells. These cells and associated cytokines/chemokines work in concert to form an immunological barrier and scavenge intrusive pathogens. In pathological conditions, however, the immunological barrier recognizes self‐antigens and destruct target cells, thus leading to autoimmune disease. On the basis of the research published hitherto, aberrantly heightened innate immune might play the role of initiator in the derailed immune function, considering that the immune system activates in a precise spatio‐temporal order (Figure 6). Under stress, DAMPs embodying melanocyte‐specific neoantigens like inducible heat shock protein 70 (HSP70i), HMGB1, S100B, and mitochondria DNA are released into the extracellular microenvironment in the form of cell debris and exosome. These DAMPs carrying danger signals further provoke innate immune activation^28^ via pattern recognition receptors (PRR) embracing TLRs, RIG‐I‐like receptors and NLRs. This process might both adversely kill melanocytes and ignite adaptive immune through generating cytokines/chemokines and antigen‐presenting.^129^, ^130^ Discussion about several critical innate immune subgroups contributing to the process is presented as followed.

Once released into the extracellular milieu, DAMPs exert an extensive effect on cell populations in the skin, such as dendritic cell (DC), NK, ILC, keratinocyte, and melanocytes per se. First, DAMPs propel the process of antigen exposure and presenting. Essentially being a ubiquitous cytoprotectant preventing the cell from undergoing apoptosis,^131^ HSP70i might enhance the delivery of melanocyte antigens to major histocompatibility complex (MHC) class II expressed on melanocytes, thus potentiating immunogenicity of the stressed melanocyte, as observed in other cell lines.^132^ Both HSP70i and HMGB1 bind to PRR on DC to beget its maturation and antigen‐presenting process.^133^, ^134^, ^135^ Second, DAMPs cultivate a proinflammatory milieu to elicit an adaptive immune response. DCs activated by DAMPs release a cascade of cytokines spanning IFN‐α, IL‐1β, and IL‐18 into the extracellular environment.^135^, ^136^ Stimulated keratinocytes also generate IL‐1β, IL‐8, CXCL9, CXCL10, and CXCL16.^133^, ^134^ This proinflammatory milieu further introduces activated NK and chemoattracted melanocyte‐specific CD8^+^T cells to melanocytes and spawns their apoptosis. Intriguingly, of the mentioned cytokines and chemokines, many are reported to be biomarkers for vitiligo like IL‐1β, CXCL9, CXCL10, and CXCL16, which might aid vitiligo diagnosis and prognosis.^81^, ^137^, ^138^ Recent proteomic studies have also proposed a panel of proinflammatory factors like lysophosphatidylcholine, platelet‐activating factor, succinic acid, CXCL4, and CXCL7 to be potential vitiligo biomarkers.^139^ There is also an elegant study integrating genomics and proteomics to define a vitiligo diseasome network, and further identified a series of potential biomarkers like CXCL12, fibronectin 1, chloride intracellular channel 1, catenin β1.^140^ Albeit these results of proteomic studies might assist vitiligo diagnosis, their mechanical implication in vitiligo pathogenesis remain enigmatic.

Whereas some melanocytes might have died even before the CD8^+^T cell arrives. HSP70‐exposed TNF related apoptosis‐inducing ligand (TRAIL)^+^DC are lytic towards TRAIL‐receptor‐expressing melanocytes.^141^, ^142^ Cells expressing TRAIL are also found to infiltrate the papillary dermis of patients with inflammatory vitiligo.^143^ Besides, upon endogenous and exogenous stress, NK, and ILC1 secret IFN‐γ to induce expression of C‐X‐C motif chemokine receptor (CXCR) 3B on melanocyte surface and release of CXCL9, CXCL10, and CXCL11 from keratinocyte and melanocyte. CXCL10 then activates CXCR3B and triggers the apoptosis of melanocytes.^130^ This result is solidified by another research illustrating that CXCR3 depleting antibodies are efficacious in preventing depigmentation.^144^

Of note, expression of “stress molecules” like MHC class I chain‐related protein A and B (MICA/MICB) also increases on DC. MICA/MICB might further activate natural killer group 2D receptor expressed by NK cells and a subgroup of cytotoxic αβ T cells like effector memory T cells (T_EM_), which might unleash their effector mechanisms, followed by melanocytes being killed and release of autoantigen from dying melanocytes.^145^

Parenthetically, a newly emergent lymphoid subclass called ILC is garnering increased attention. The ILC family is a heterogeneous population of non‐B non‐T lymphocytes encompassing ILC1, ILC2, ILC3, and, possibly, regulatory ILC subclasses.^146^, ^147^ They share many similarities with T cells and are even suggested to be the innate counterparts of T cell subsets. ILC family manifests great complexity due to their subgroup interconversion and heterogeneity between individuals.^147^ Interestingly, their residency in nonlymphoid tissues in steady state and rapid response under stimulation are somehow akin to T_RM_.^148^ And their implication in killing melanocytes still merits more detailed analysis.

Here we can conclude that deranged innate immunity paves the road for the coming of further adaptive immunity by creating a local inflammatory milieu and destructing a minority of melanocyte to release melanocyte‐specific antigens.

A cessation of the abnormal innate immunity is urgently needed as a part of vitiligo management, unfortunately, novel strategies to accomplish the goal are few. Contributions of Mosenson et al.^149^ are the mainstay in the development of strategies for innate immunity‐normalization in vitiligo. In 2013, the team switched one amino acid in the sequence of HSP70i through a DNA vaccine, and the mutated HSP70i prevented the activation of DC into inflammatory status thus abrogated the concomitant antigen‐specific T cells accumulation.^149^ They successfully reversed the depigmentation in mice with spontaneous vitiligo. In 2018, similar results were acquired in Sinclair swine.^150^ And now the team is planning for administering the gene‐based approach in patients, which might induce long‐term repigmentation of vitiligo lesions. As for pharmacotherapy, topically applied vitamin D is reported to reduce the number of DC in the vitiligo skin.^151^ Besides, in psoriasis, vitamin D also inhibited the function of DC.^152^ But the efficacy of vitamin D in vitiligo treatment still warrants clarification. And the phototherapy, NB‐UVB might also be associated with decreased Langerhans cell distribution in vitiligo epidermis after treatment.^153^

Apart from what we have discussed, IL‐27 was also reported to act on DC to suppress the T cell function, thus employing a DNA vaccine to topically overexpress IL‐27 is also therapeutically exploitable.^154^ However, members functioning in the innate immune system are not restricted to DC, and pharmacological exploration on regulating NK cell and other cells might also be concerned.

## 6 ADAPTIVE IMMUNE ACTIVATION

Distinguished from innate immune activation, adaptive immunity is characterized by giving antigen‐specific responses and memorizing antigens. In vitiligo, adaptive immune activation is responsible for killing melanocytes specifically, leaving other cells in the vicinity untouched (Figure 6). It's also a major player in melanocyte destruction in vitiligo. In this part, we mainly talk about T cells rather than B cells, as it remains an open conundrum whether B‐cell‐mediated humoral immunity participates in vitiligo pathogenesis.

### 6.1 Executioner: CD8^+^T cell

In cell‐specific autoimmune diseases, such as multiple sclerosis, rheumatoid arthritis, and vitiligo, CD8^+^CTL has always been underpinned as a fundamental role in disease progression.^155^, ^156^ The notion that CD8^+^T cell serves as the last procedure in the process of extensive melanocytes destruction and confers the final and fatal onslaught to melanocytes in the pathogenesis of vitiligo is widely accepted. In fact, the character of CD8^+^T cell as the executioner in vitiligo was gradually elucidated by a multitude of elegant experiments as follows. Higher serum frequency of melanocyte‐specific CD8^+^T was observed in vitiligo patients compared to their healthy counterparts.^157^ Additionally, their frequency is related to disease severity.^28^ Patchy infiltration of T cells was found adjacent to melanocytes, especially in the leading edge of vitiligo depigmentation.^158^, ^159^, ^160^ The proportion of T cells in this area is also CD8^+^T‐skewed, as manifested by elevated CD8^+^T cell number and decreased CD4/CD8 ratio.^160^ Isolated T cells from biopsies exhibited autoreactivity to a series of melanocyte‐specific antigens, such as melan‐A, gp100 and tyrosinase, and cytotoxicity to autologous melanocytes in vitro.^161^, ^162^ The depletion of CD8^+^T cells abrogates melanocyte destruction, however, enrichment for these cells aggravates it.^161^ Recently, programmed cell death protein (PD)‐1 and T‐cell immunoglobulin and mucin‐domain containing‐3 molecules, which serve as immune checkpoints to mediate braking of cytotoxic response, are found to be highly expressed on vitiligo CD8^+^T cells. This is surmised to be a compensation mechanism of the immune system to keep peripheral tolerance towards self‐tissue, in face of hyperactivated CD8^+^T cells.^163^ A study administrating programmed death‐ligand 1 on vitiligo mice successfully reversed the depigmentation also endorses the participation of immune checkpoint in self‐immunoresponse against melanocytes.^164^

Mechanically, CD8^+^T cell mediates melanocyte apoptosis in various ways. Secreting perforin/granzymes and applying Fas–FasL mechanisms are the two most frequently mentioned.^165^ But differing views disputed about the contribution of the two mechanisms to melanocyte destruction in vitiligo. Research based on a mouse model with DNA immunization against a xenogeneic form of TRP‐2 revealed that autoimmunity against normal melanocytes required perforin but Fas–FasL was dispensable.^166^ Of the other three studies, depending on transgenic mice with tyrosinase‐specific T cell clone,^167^ transgenic mice carrying T cells with an HLA‐A2 restricted human tyrosinase reactive T cell receptor (TCR)^168^ and mice manifesting melanoma‐induced autoimmune symptoms (vitiligo‐like white coat),^143^ respectively, the former two regard perforin as an unnecessary mechanism in melanocyte destruction. And the first and third affirmed the implication of the Fas–FasL system in killing melanocyte. The inconsistency among these research probably results from the heterogeneity of modeling methods. And these vitiligo models are simulating different pathophysiology of distinct genetic background and risk factors. Thus, the four viewpoints might not exclude each other in vitiligo pathogenesis. The contrasting mechanisms employed in transgenic or self‐antigen challenged cytotoxic‐T‐cells‐induced melanocyte destruction also merit further exploration. Moreover, IFN‐γ released by CD8^+^T cells also dictates the apoptosis of melanocytes in multifaceted ways. First, the IFN‐γ‐CXCL9/CXCL10‐CXCR3 axis further collects more CD8^+^T cells and promotes their effector function, leading to a severe immune response.^122^, ^144^, ^169^, ^170^ Another study established the role of IFN‐γ in increasing the ratio of Treg versus effector T cells.^168^ Second, a transient pulse of IFN‐γ may provide a microenvironment of locally high concentrations at which IFN‐γ exerts a cytotoxic effect on melanocytes precisely and efficaciously, leaving other cells in the vicinity uninfluenced.^171^ Third, IFN‐γ generated by CD8^+^T might impair cysteine uptake and trigger ferroptosis in tumor cells as recently reported,^121^ whether this mechanism operates in IFN‐γ‐associated melanocyte destruction in vitiligo remains to be clearly delineated.

To conclude, extensive studies having lent credence to the role of executioner played by CD8^+^T cell in vitiligo, researchers have shifted their interest to explore activation, chemotaxis, suppression, homing, residency, and survival of CD8^+^CTL, exactly the part we will expound later.

### 6.2 Alarm sensor and cytotoxic killer: Tissue‐resident memory T cells (T_RM_) cells

Vitiligo lesions are reversible in the course of the disease. About 50% recurrences take place at the same location within one year after discontinued treatment,^172^ this provides some hint as to a memory component of autoimmunity in vitiligo. Memory T cells, especially T_RM_ cells, are proposed to be the culprit underlying this “skin memory disease.”^173^

According to the expression of the homing receptors and migratory patterns, memory cells protecting human skin are parsed into four subgroups, which were labeled central memory T cells (T_CM_), T_EM_, migratory memory T cells, and T_RM_.^174^, ^175^ Canonical surface markers of CD69 (a T cell activation marker), CD103 (integrin α_E_) and CD49a (α‐subunit of the α1β1 integrin receptor) are frequently utilized to distinguish epidermal T_RM_ from circulating memory cells. In healthy adult human skin, most T_RM_ cells are CD103^−^CD4^+^ and reside in the dermis. While CD103^+^T_RM_, both CD4^+^ and CD8^+^, are enriched in the basal layer epidermis.^176^ Skin T_RM_ cells are large in number, but variable among individuals.^177^ Of the estimated 2.0 × 10^10^ resident T cells in the entire skin surface,^178^ skin T_RM_ occupies a large fraction, with the proportions vary between 20% and 60%.^175^ Assuming a protective role in normal organisms, T_RM_ appears after the resolution of skin inflammation, persists for a long time in tissue, and is endowed with the propensity for more rapid response to invasive pathogen or tumor cells than circulatory memory cells.^172^, ^179^, ^180^, ^181^ Aside from eliminating pathogens, when aberrantly activated and sensitized to harmless self‐antigens, nonetheless, T_RM_ might contribute to autoimmune diseases.^182^ T_RM_ is also long‐lived. Because the role of CD4^+^T_RM_ is still ill‐defined in vitiligo, we discuss only CD8^+^T_RM_ in this part.

Lack of recirculation is a characteristic feature of T_RM_, and the mechanism for its residency in the skin is being gradually unveiled. T_RM_ residency is a reciprocal interaction between T_RM_ endogenous motivating factors and the microenvironment. T_RM_ has to adapt to local survival cues, tethering within the skin and ignore egress signals.^183^ CD103 is the α‐subunit of the α3β7 integrin receptor which enables T_RM_ to anchor to epithelial cells by binding to E‐cadherin. However, binding to E‐cadherin is dispensable for T_RM_ residency,^71^ though T_RM_ cells expressing CD103 are more potent in generating cytokines.^174^ Sphingosine1‐phosphate receptor 1 is required for lymphocyte egress from peripheral tissues, intriguingly motivated by transcription factor Kruppel‐like factor 2, and CD69 enhances its internalization and degradation thereby maintaining T_RM_ residency in the skin.^184^ CD8^+^T_RM_ in the skin also requires the aryl hydrocarbon receptor for long‐term survival.^185^ To facilitate T_RM_ conversion, differentiation, development and residency, tissue also forms appropriate cytokine niches embodying IL‐12, IL‐15, IL‐18, IL‐33, IFN‐β, TNF‐α, and transforming growth factor (TGF)‐β.^184^, ^186^, ^187^, ^188^ Among the cytokines, TGF‐β induces expression of CD103,^189^ and IL‐15 supports T_RM_ development, maintenance, and survival.^188^ Reliance of T_RM_ on IL‐15 is substantiated by several studies, for example, melanocyte‐specific T_RM_ cells cluster in high IL‐15 production sites like hair follicles in the vitiligo skin,^190^ and antibody blockade of IL‐15 signaling durably reverses depigmentation.^181^

As an alarm sensor, T_RM_ alone is insufficient for inducing and maintaining depigmentation though possessing cytotoxicity. Ensuing melanocyte‐specific antigen stimulation, CD8^+^T_RM_ secrets IFN‐γ, which proves to be apoptosis‐inducing.^171^ CD49a^+^CD8^+^T_RM_ are even poised for cytotoxic responses by producing perforin and granzyme B.^176^ CD8^+^T_RM_ residing around hair follicular might also curtail the entry of melanocyte precursors from the follicular reservoir to vitiligo lesions, thus conferring disease flares or repigmentation blockade.^175^ But the seminal research by Richmond et al.^191^ solidified that T_RM_ must work in concert with recirculating T cells in vitiligo pathogenesis, through depleting recirculating T_CM_ or inhibiting their recruitment to the skin to successfully reverse vitiligo. Vigorously secreted IFN‐γ is also recruiting T_CM_ and B cells to the sites T_RM_ resides in, presumably by upregulating the production of downstream chemokines and vascular cell adhesion molecule 1.^191^, ^192^ Similarly, CD49a^−^CD8^+^T_RM_ also manifests its robust competence in recruiting other immune cells via IL‐17 production.^176^

Deranged T_RM_ is a critical component of the overridden natural immunity intertwined in the development of vitiligo. Accordingly, we summarized T_RM_ hallmarks and residency along with their functions in melanocyte destruction in the hope of illuminating novel therapies to tip the balance towards autoimmune suppression or tolerance. Nevertheless, methods to fundamentally deplete T_RM_ from the skin are still far from a definite therapeutic effect.^181^, ^193^ Considering the effect of T_RM_ in countermining infectious pathogen and tumor cell challenge, an in‐depth understanding of T_RM_ must be obtained before manipulating them in therapeutic approaches.

### 6.3 Incompetent suppressor: Treg

Tregs contribute to immune homeostasis by suppressing immune activation and maintaining peripheral self‐tolerance. They enforce a negative effect on the activation and expansion of autoreactive CD4^+^ or CD8^+^ T cells.^194^, ^195^ That melanocyte‐specific CD8^+^ T cells can be detected in healthy people without vitiligo suggests autoimmune kept in check, but in vitiligo, the checkpoint is invalid.^196^ The sparse Treg found in vitiligo skin is also indicative of autoreactive CD8^+^ T cells' unopposed action.^197^

The transcription factor Forkhead box P3 (FoxP3) remains the most reliable Treg marker. Apart from FoxP3, Tregs are also known to highly express CC chemokine receptor (CCR) 4 and CCR7, both of which mediating Tregs homing to tissues together with their ligands CCL21 and CCL22.^194^ CD25, cytotoxic T lymphocyte‐associated antigen‐4, PD‐1, and inducible T‐cell costimulator are also expressed on Treg surface as suppressive molecules.^198^ Treg employs mainly two mechanisms in suppressive function, namely, cytokine secretion (TGF‐β1 and IL‐10) and cell–cell contact. Also, Tregs reportedly restrain melanocyte‐specific CD8^+^ T cells by inducing their anergy and dampening their proliferation and cytokine production (IFN‐γ, TNF‐α, and IL‐2).^199^ Tregs‐orchestrated downregulation of costimulatory molecules CD80 and CD86 on antigen‐presenting cells contributes to the process of suppression as well.

Extensive reports have delineated the landscape of disequilibrium between hyperactivated autoreactive CD8^+^ T cells and incompetent suppressive Tregs, with some displaying contradictory results. But research having effectively found the underlying cause of the incapable immunosuppression are few. Numbers and proportion of serum and skin Tregs are found to be significantly decreased in a series of reports^195^, ^200^, ^201^ when comparing vitiligo patients with healthy controls. Intriguingly, some studies demonstrated unaltered or even elevated numbers of Treg in vitiligo skin.^196^, ^202^, ^203^ Discrepancies between studies about counts of Tregs might be attributed to (1) each sampling reflects merely a cross‐section of a developing disease progression, thus the panorama of changing Treg counts can't be obtained; (2) many other activated CD4^+^ cells also express FoxP3 and FoxP3 antibodies employed in the experiment can also bind to them^204^; (3) racial and age factors.^194^ Several studies illustrated the notably decreased levels of TGF‐β^195^, ^201^, ^205^ and IL‐10^205^, ^206^ in the serum of vitiligo patients in contrast to controls. FoxP3 expression in serum and lesional skin,^201^ FoxP3 mRNA expression in lesional and perilesional skin declined in vitiligo patients.^207^ The two points indicate functional deficiencies in Tregs derived from vitiligo patients, though only a few studies tried to find the underlying pathomechanism. Klarquist et al.^196^ attributed sparse Tregs in vitiligo skin to reduced expression of CCL22‐induced Treg homing failure. Zhang et al.^195^ ascribed Treg incapability to a diminution of functional modulator HO‐1, and applying HO‐1 agonist restored immunoregulatory Treg function probably by increasing IL‐10 and latency‐associated peptide expression.^195^ A subset of Treg maintaining in tissue for relatively long periods termed memory Tregs (mTregs) is also found to be dysfunctional in psoriasis. And the contribution of mTregs to vitiligo pathogenesis remains to be elucidated in the future.^208^

Tregs deficiency‐induced unrestricted autoimmune response culminates in melanocyte death in vitiligo pathogenesis. Therefore, the analysis above might contribute to the clarification of mechanisms underlying incompetent Tregs and melanocyte destruction.

### 6.4 Probable booster: T helper cells

Early studies found that CD4^+^ T helper (Th) cells infiltrating the perilesional skin are type‐1‐skewed,^209^, ^210^ with their proportion reaching approximately 95%.^178^ This leads to enhanced production of inflammatory cytokines like IFN‐γ and TNF‐α.^211^ Moreover, keratinocytes respond to Th1 cytokines by eliciting the migration of Th1 and to Th2 cytokines by recruiting both Th1 and Th2 cells.^212^ This imbalanced sensitivity to Th1‐ and Th2‐ cytokines might partly explain the Th1‐ polarization in vitiligo skin. CD4^+^ T cells are also involved in melanocyte loss by destroying MHC class II expressing cells via Fas–FasL interaction, and melanocytes exactly express both MHC class II and Fas.^213^ The participation of Th2 in vitiligo pathogenesis has not been validated, though Th2‐produced IL‐4 can inhibit melanogenesis directly in vitro.^214^

Another subgroup of T‐helper cells, Th17, together with its cytokine IL‐17 is implicated in the etiology of several autoimmune diseases, psoriasis, rheumatoid arthritis, multiple sclerosis, and primary Sjogren's syndrome included.^215^ And its role remains to be further defined in vitiligo though progress has been obtained. Th17 has been found to infiltrate the vitiliginous skin.^129^ They diffusely infiltrate the upper dermis, hence Th17 cells putatively act on melanocytes through cytokines secretion instead of direct interaction.^129^ A wealth of studies has illustrated that levels of serum Th17,^216^, ^217^ serum IL‐17,^80^, ^216^, ^218^, ^219^ lesional tissue IL‐17 mRNA,^80^, ^220^ perilesional skin IL‐17A and IL‐17A receptor^129^ are all relatively higher in vitiligo patients. That NB‐UVB light treatment could decrease lesional and perilesional IL‐17 expression accompanied by a reduction in Vitiligo Area Scoring Index (VASI) reiterates Th17‐participation in vitiligo.^207^ Singh et al.^215^ reviewed that serum IL‐17 levels are positively correlated with the extent of body area involvement, VASI and Vitiligo Disease Activity. Accordingly, Th17 and IL‐17 might participate in the pathomechanism of vitiligo given the evidence enumerated above.

Mechanistically, the capacity for melanogenesis and antiapoptosis could be dampened by IL‐17 in melanocyte in vitro.^129^ It should be highlighted that Zhou et al.^58^ demonstrated IL‐17‐stimulated melanocytes experience apoptosis because of mitochondrial dysfunction and ROS accumulation. This result directly connects IL‐17 to melanocyte demise. Besides, in a study of adoptive cell transfer therapy in melanoma, the authors illustrated that Th17‐polarized CD4^+^ cells confer antitumor efficacy and concomitant autoimmune vitiligo, despite the efficacy is surprisingly IFN‐γ‐dependent.^221^ Studies of Speeckaert et al.^222^, ^223^ provide insights into a new paradigm of Th17 in vitiligo. They showed that Th17.1 cells instead of Th17 cells are increased in vitiligo. As a subset of Th17 cells, Th17.1 cells secret both IL‐17 and IFN‐γ and gradually differentiate into nonclassical Th1 cells. And there is a delicate Th17/Th17.1/Th1 balance which is altered significantly in the disease progress.

Although a series of publications have underpinned the putative role of Th17 in melanocyte destruction, outcomes of therapy pertinent to Th17 still could not meet people's anticipation. For example, secukinumab (IL‐17A inhibitor) application leads to new depigmentation onset in patients rather than halt vitiligo development in the clinical trial.^223^ This indicates that the effect of Th17 should be properly understood, especially in the context of complex plasticity of Th17 encountering different environmental triggers.^224^ And deeper insights into immune mechanisms might instigate an ongoing discussion about the role of Th17 in vitiligo.

### 6.5 Possible interventions to retrieve adaptive immune anomalies

Immunosuppressive therapies have always been the mainstay of vitiligo treatment, which is echoed by the profound effect of immunology anomaly on melanocyte destruction. For now, the recommended immunosuppressive interventions include topical steroids, calcineurin inhibitors (tacrolimus and pimecrolimus), and oral pulse steroids. But these interventions are just moderately effective and financially burdensome. Fortunately, researchers have been translating basic research into target therapies. And in this part, we will discuss state‐of‐the‐art strategies for targeted immunomodulation in vitiligo.

As previously mentioned, IFN‐γ‐CXCL9/CXCL10‐CXCR3 axis is indispensable in the killing of melanocytes by the CD8^+^ T cells, and compromising the axis was effective in halting vitiligo progression and facilitating repigmentation.^122^, ^144^, ^225^ Mediated by Janus kinase (JAK) 1 and 2, in this axis, keratinocyte senses IFN‐γ and generates CXCL9/CXCL10.^122^ Rosmarin et al.^226^ focus on the research of JAK inhibitors in vitiligo treatment, which is of high clinical relevance and therapeutic significance. Their prospective, randomized, vehicle‐controlled phase‐2 trial revealed that JAK inhibitor ruxolitinib was effective as monotherapy in vitiligo treatment, without treatment‐related serious adverse events reported.^226^ However, this therapeutic option must be sustained to protect from disease relapses. That JAK inhibitors might just abrogate chemotactic approach of cytotoxic cells instead of dislodging the T_RM_, which recruits cytotoxic cells to the skin, might explain the failure in generating durable treatment responses.^193^

Referring to T_RM_, it might be a culprit manipulating vitiligo relapses. Their continuous recruitment of autoreactive cytotoxic T cells from circulation by secreting IFN‐γ should be targeted to realize durably vitiligo reverse. Richmond et al.^181^ has successfully depleted T_RM_ from vitiligo lesional skin through long‐term administration of the anti‐CD122 antibody. CD122 is a subunit of the heterotrimeric IL‐15 receptor, thus, CD122 blockade might impair the residency of T_RM_ in the skin by nullifying the support from IL‐15.^181^ Besides, T_RM_ exhibits unique metabolic panorama compared to effector T cells and other memory T cells. T_RM_ relies on exogenous free fatty acid (FFA) uptake to maintain its residency in the skin. Accordingly, it might be promising to intercept the critical metabolism pathway to deplete T_RM_, for example, trimetazidine, which blocks mitochondrial β‐oxidation of FFA to decrease the longevity of T_RM_ in the skin.^227^, ^228^

Treg is endowed with the immunosuppressive properties, hence it is always acclaimed as “living drugs.” In therapeutic strategies for vitiligo, “quantitively” or “qualitatively” potentiating Treg might be an optimistic option due to its endogeneity. First, Treg expansion is an efficient strategy. IL‐2 is fundamental for the differentiation, survival, and function of Treg.^229^ Moreover, Treg expresses the high‐affinity IL‐2 receptor to collect IL‐2 from the microenvironment. Thus IL‐2 therapy is speculated to be efficacious in Treg expansion. Low‐dose IL‐2 treatment has exhibited efficacy in alopecia areata,^230^ systemic lupus erythematosus,^231^ type 1 diabetes,^232^ and hepatitis virus C‐induced vasculitis.^233^ Besides, TNF receptor 2 (TNFR2) is highly expressed on Treg compared to other T cells, and using specific antibodies or agonists to stimulate TNFR2 is reported to efficiently expand natural Tregs in graft‐versus‐host disease.^234^ Furthermore, targeting the Notch‐1 signaling pathway reportedly enhance Treg populations and suppressive function,^235^ which is pharmacologically exploitable. Another possible option is adoptive Treg therapy, which is harvesting Treg from the patient's circulation to expand them in vitro, and transfer the expanded Tregs back to the patient. But the scarcity of Tregs in the blood and slow rate of expansion in vitro are the major constraints for the clinical application.^236^ Alternative to Treg expansion, elevating the recruitment of Treg to the epidermis through gene‐gun‐treatment‐induced CCL22 overexpression also delayed vitiligo progression.^237^

Second, “qualitatively” edited Treg has been much heralded in recent years. Antigen‐specific Tregs accumulate in targeted tissue and function 1,000– to 10,000‐ fold higher than the nonspecific “bystander” Tregs. It is, therefore, tempting to speculate investing a pool of antigen‐specific Treg into one treatment. Approaches like genetically engineered Treg cells expressing chimeric antigen receptor (CAR) or artificial TCR enable targeting specific antigens of interest. With the help of CAR and TCR technology, abundant antigen‐specific Tregs can be acquired through in vitro expansion.^238^ Apart from adding specificity to Tregs, T cells with specificity can be transformed into Tregs. The effector T cells could portray the immunosuppression of Treg after lentiviral‐based overexpression of FoxP3.^239^ But the transduced Treg is at the risk of instability that may still function as effector T cells. Additionally, mesenchymal stem cell is being developed to exert their broad immunosuppressive capacity on autoimmune diseases.^240^ Limitations still exist, however, considering the current processes of Treg manufacturing are costly and labor‐intensive. Multiple obstacles must be circumvented before these technologies are applied in vitiligo, in light of the low‐priority of vitiligo in global health despite its impact on patients’ psychological health.

An objective assessment method of therapeutic efficacy is necessary in vitiligo management, in addition to standard photographing protocol^241^ and helpful instruments (Wood's lamp, dermoscopy, and in vivo confocal microscopy). To meet the clinical needs, several studies employed imaging analysis to evaluate the changes of the depigmentation area, but restricted by factors like large depigmentation, curved body surface and complex repigmentation patterns.^242^, ^243^, ^244^, ^245^ A recent publication introduced a delicate method to generate VASI automatically, through superpixel‐generated computerized digital image analysis of clinical photographs with ImageJ.^246^ Another study proposed a protocol for calculating the true depigmentation area which could serve as a “gold standard” for validation of other digital tools.^247^ The combination of these two methods might facilitate both vitiligo diagnosis and therapeutic efficacy assessment.

## 7 CONCLUSIONS

In the context of genetic vulnerability, ROS is a crucial player in the initiation of vitiligo. ROS contribute to the melanocyte destruction in the starting stage from many aspects, like DNA damage, lipid peroxidation, mitochondrial dysfunction and endoplasmic reticulum stress. Besides, investigations into the death modalities like necroptosis, pyroptosis, ferroptosis, oxeiptosis, and parthanatos should extend from tumor research to melanocyte death studies in vitiligo to discover new vitiligo therapies. Derailed immunity is a central factor in melanocyte destruction in vitiligo. Innate immunity paves the road for adaptive immunity response through generating inflammatory microenvironment and antigen‐presenting. In the adaptive immunity, Treg fails to suppress the autoreactive immunity, T_RM_ works in concert with CD8^+^CTL to kill melanocytes with support of Th. Besides, measures to potentiate the suppressive capability of Treg or scavenge dysfunctional T_RM_ specifically to rebuild the self‐tolerance in vitiligo remain further development.

From environmental stimulus to ROS‐induced damage and autoimmunity, the program of killing melanocytes is activated in a roughly hierarchical fashion in vitiligo pathogenesis, as depicted in Figure 5. The figure might help to better understand the mechanisms of vitiligo onset and development, and the blank representing unknown factors underlying the melanocyte destruction needs to be fulfilled by further studies.
